# Possible Economies with Steam Boilers

**Published:** 1921-04-23

**Authors:** 


### HOSPITAL ENGINEERING REFORMS.

### Possible Economies with Steam Boilers.

Every modern hospital has a steam boiler; sometimes the steam is employed to furnish power for driving dynamo machines, laundry machinery, and others; but the greater portion of it is used for heating the building, and for providing a supply of hot water. In the past the question of the economical working of the boiler has not come up; it has not been so much considered as keeping a supply of hot water, and a proper temperature in different parts of the building. Now, when hospitals are coming on to bad times, the steam boiler is a place where economy must be rigorously enforced.

One of the points that has been overlooked in the working of boilers in the past has been the question of obtaining the right temperatures at different parts of the steam system. There is a very old law, due to the French physicist Carnot, which may be given in a modified form, that the economy of any heat engine depends upon the range of temperature that is usefully employed. The boiler is essentially a heat engine; it may be considered as two heat engines if preferred, for that method of dealing with the problem simplifies the treatment. The two heat engines are, first, the furnace in which the coal is burnt, with its accessories the flues through which flow the hot gases formed by the combustion of the coal; second, the water and steam portions. The two parts of the boiler—that in which the coal is burnt, and that in which steam is generated—are quite distinct from each other; they are separated from each other everywhere by metal diaphragms, across which the heat from the hot gases passes to the water and the steam. Therefore, they may be quite correctly considered as two distinct heat engines.

On the other hand, the whole steam system, the boiler furnace and flues, the water and steam space in the boiler, the steam pipes, the steam engine, the radiators, the calorifiers, or whatever may be employed to deliver the heat of the steam to water, or any other substance that is required to be heated, may be considered as a single heat engine. In either case the range of temperature that is usefully employed marks the limit of the efficiency of the system. This means that the higher the temperature at which the heat engine starts, and the lower the temperature to which it works, the greater is the economy.

### Applying Carnot's Law to the Boiler Furnace.

When coal is burnt in the boiler furnace, the gases that are produced by combustion are raised to a high temperature, and this temperature marks one of the limits of economy. Before economy was studied a temperature of 2,000° F. was usually the highest contained at the furnace itself; and, on the other hand, the hot gases were delivered at the base of the chimney often at a temperature of 1,000° F. This means that only 1,000° F., or 50 per cent. of the total range of temperature, was usefully employed, and therefore the highest efficiency theoretically obtainable in the boiler furnace was 50 per cent. Against this there were always other losses, such as the leakage of air into the boiler flues, the partial stoppage of the draught by clinker on the grate bars; and the efficiency of the boiler as a whole was necessarily less than 50 per cent., because the efficiency of the steam and water side had to be equated with that of the furnace portion. Hence the combined efficiency might easily be in the neighbourhood of 30 to 35 per cent. Even now, when attention has been given to the subject, measurements that have been taken upon boilers in use show that efficiencies of only 35 to 55 per cent. are common, while it should be perfectly practicable to obtain an efficiency of at least 75 per cent.

### The Modern Boiler.

In the modern boiler the furnace temperature has been raised to 3,000° F., while the hot gases are delivered at the base of the chimney at 300° F., and in some cases at 200° F.; in one type of boiler a temperature of 3,500° F. has been obtained in the boiler furnace. Taking the case of the furnace having a temperature of 3,000° F., and the temperature of the gases delivered to the chimney at 300° F., the possible furnace efficiency is raised to 90 per cent.; and if the efficiency of the steam side of the boiler is also raised to 90 per cent., the combined efficiency of the boiler as a whole is raised to 81 per cent. This means, of course, decreased coal consumption, decreased capital cost, and decreased labour.

### How to Ensure the Proper Temperatures.

The first difficulty that arises is how to ensure that the high temperature mentioned shall always be present in the boiler furnace, and the low temperature at the base of the chimney; and the answer is by the constant use of measuring instruments, of thermometers specially designed for the purpose. The electric thermometer and pyrometer that will be described in the next article enable this to be done.

